# Effects of inhaled nitric oxide on outcome after prolonged cardiac arrest in mild therapeutic hypothermia treated rats

**DOI:** 10.1038/s41598-018-25213-1

**Published:** 2018-04-30

**Authors:** Anne Brücken, Christian Bleilevens, Philipp Berger, Kay Nolte, Nadine T. Gaisa, Rolf Rossaint, Gernot Marx, Matthias Derwall, Michael Fries

**Affiliations:** 1 Department of Intensive Care Medicine, Medical Faculty, RWTH Aachen University, Pauwelsstr. 30, 52074 Aachen, Germany; 2 Department of Anaesthesiology, Medical Faculty, RWTH Aachen University, Pauwelsstr. 30, 52074 Aachen, Germany; 3 Institute of Neuropathology, Medical Faculty, RWTH Aachen University, Pauwelsstr. 30, 52074 Aachen, Germany; 4 Institute of Pathology, Medical Faculty, RWTH Aachen University, Pauwelsstr. 30, 52074 Aachen, Germany; 5 Department of Anaesthesiology, St. Vincenz Hospital Limburg, Auf dem Schafsberg, 65549 Limburg, Germany

## Abstract

Guidelines endorse targeted temperature management to reduce neurological sequelae and mortality after cardiac arrest (CA). Additional therapeutic approaches are lacking. Inhaled nitric oxide (iNO) given post systemic ischemia/reperfusion injury improves outcomes. Attenuated inflammation by iNO might be crucial in brain protection. iNO augmented mild therapeutic hypothermia (MTH) may improve outcome after CA exceeding the effect of MTH alone. Following ten minutes of CA and three minutes of cardiopulmonary resuscitation, 20 male Sprague-Dawley rats were randomized to receive MTH at 33 °C for 6hrs or MTH + 20ppm iNO for 5hrs; one group served as normothermic control. During the experiment blood was taken for biochemical evaluation. A neurological deficit score was calculated daily for seven days post CA. On day seven, brains and hearts were harvested for histological evaluation. Treatment groups showed a significant decrease in lactate levels six hours post resuscitation in comparison to controls. TNF-α release was significantly lower in MTH + iNO treated animals only at four hours post ROSC. While only the combination of MTH and iNO improved neurological function in a statistically significant manner in comparison to controls on days 4–7 after CA, there was no significant difference between groups treated with MTH and MTH + iNO.

## Introduction

Sudden cardiac arrest (CA) remains one of the leading causes of death worldwide^1^. Despite improvements in pre-hospital care, mortality rates of out-of-hospital CA (OHCA) victims are still high^2,3^. Up to 60% of the survivors suffer from moderate to severe cognitive deficits 3 months after resuscitation^4,5^. These poor outcomes are mainly due to the post-CA syndrome^6^, including cerebral and myocardial dysfunction^7^ subsequent to a pronounced inflammatory response^8^.

Mild therapeutic hypothermia (MTH) is the only proven treatment to reduce neurological sequelae and mortality after CA^9^.

However, a large population of CA victims does not seem to profit from hypothermia and may require additional treatment^10^. Currently, no pharmacological agent is available to further improve outcomes for CA victims beyond targeted temperature management (TTM) (32–36 °C), although some promising results have been reported using inhaled xenon^11^.

Initially developed as a selective pulmonary vasodilator, inhaled nitric oxide (iNO) has been claimed to exert systemic effects without causing systemic vasodilation^12^. In the setting of ischemia- and reperfusion-injury (I/R), iNO attenuates myocardial injury in mice^13^ and swine^14^, and reduces hepatic injury in patients undergoing liver transplantation^15^. Additionally, iNO might play a crucial role in brain protection by preventing the early induction of TNF-α and IL-6 expression in the brain via soluble guanylate cyclase -dependent mechanisms^16^. We recently reported that nitric oxide inhalation started during cardiopulmonary resuscitation (CPR) improves clinical neurological outcomes after prolonged cardiac arrest in rats and swine^17,18^. However, these studies did not pursue the comparison to MTH.

A recent study from Kida and colleagues on the role of nitric oxide synthase (NOS3) in hypothermia-mediated neuroprotection showed that the absence of NOS3 abolished hypothermia induced brain protection in NOS3^−/−^ mice post CA and CPR. An effect that could be abolished with iNO in this model^19^.

Here we examined the effects of short term iNO inhalation one hour after return of spontaneous circulation (ROSC) following prolonged CA in hypothermia treated rats. We hypothesized that iNO combined with MTH improves cerebral and myocardial outcomes exceeding the effect of MTH alone.

## Methods

The study protocol was approved by the appropriate federal body (Landesamt für Natur, Umwelt und Verbraucherschutz Nordrhein-Westfalen; Recklinghausen; Germany) and follows the Guide for the Care and Use of Laboratory Animals by the National Research Council (National Academies Press, 1996) and the ARRIVE guidelines (National Centre for the Replacement, Refinement and Reduction of animals in research, 2010). Furthermore, all data and outcomes are presented in accordance with the Utstein style guidelines for uniform reporting of laboratory CPR research^20^.

The experiments were carried out in 20 male Sprague-Dawley rats (Charles River, Sulzfeld, Germany) weighing between 400–500 g using an established rodent CA model^21^. Animals were housed under standardized conditions, including adequately spaced cages (60 cm × 40 cm; type 2000; Tecniplast; Buguggiate; Italy) and a 12-hour light-dark cycle. Animals free access to water and food prior to the study was guaranteed.

MTH at 33 °C, initiated immediately after ROSC and maintained for 6 hours (MTH, n = 6) was tested against 20ppm iNO (1 hour after ROSC for 5 hours) augmented MTH (MTH + iNO, n = 7) and no treatment (Control, n = 7). Primary endpoint was the neurological outcome compared between groups.

### Animal preparation

We used a rat model of CA and CPR as previously described^21^: On the experimental day, rats were anaesthetized with an intraperitoneal injection of pentobarbital (45 mg · kg^−1^). In case signs of animals’ discomfort were noted (i.e. sudden rise in heart rate (HR), respiratory rate, or tail or paws movement), additional doses (10 mg · kg^−1^) of pentobarbital were administered. Prior to the placement of the rat on a surgical board in supine position, the animals’ chest and back were shaved completely, to enable direct skin-contact of the defibrillator electrodes used for defibrillation during CPR.

Afterwards, a modified 14 G cannula (Abbocath-T, Abbott Hospital Division, North Chicago, IL, USA) was used for oral intubation of the trachea, to enable mechanical ventilation of the animals, using a specialized ventilator for precise delivery of NO (Servo Ventilator 300 A, Siemens, Munich, Germany) with a FiO_2_ of 0.3. End-tidal pCO_2_ was continuously monitored with an infrared CO_2_ analyzer (Cap Star 100, CWE Inc., Ardmore, PA, USA) and kept between 35 and 40 mmHg by adjusting the respiratory frequency. Monopolar needle electrodes (MLA1204 Needle Electrodes, ADInstruments, Oxford, UK) were used for continuous three lead electrocardiogram derivation. A back-coupled heating pad (TCAT-2LV-controller, Physitemp Science Products, Hofheim, Germany) was used to monitor and maintain the body temperature between 37.0–37.5 °C. The left femoral blood vessels were surgically exposed; the left femoral artery was cannulated with a polyethylene catheter (PE 50) and connected to a high sensitivity transducer (Capto SP 844 Physiologic Pressure Transducer, Capto Inc., Skoppum, Norway) for the measurement of mean and diastolic arterial pressures (MAP and DAP). The left femoral vein was cannulated with an additional PE 50 catheter for administration of fluids and epinephrine during CPR. All catheters were flushed intermittently with saline solution containing 2.5 IU · ml^−1^ of heparin.

### Experimental procedure

Using the sealed envelope method, animals were randomly assigned into three groups: Animals received either MTH initiated immediately after ROSC and maintained for six hours (MTH, n = 6), 20 ppm iNO (for five hours starting one hour post ROSC) augmented MTH (MTH + iNO, n = 7) or normothermia and ventilation with an FiO_2_ of 0.3 (Control, n = 7).

Ventricular fibrillation (VF) was induced by transoesophageal electrical stimulation, using an electrode, placed via fluoroscopy guidance. A commercially available fibrillator (Fi 20 M, Stockert GmbH, Freiburg, Germany) was used to deliver alternating current (10 V, 50 Hz) to the heart, leading to VF. The abrupt decrease in MAP to less than 20 mmHg confirmed CA; ventilation was stopped simultaneously. After 10 minutes of untreated CA, CPR was initiated via chest compression, using a custom made mechanical thumper at a stroke rate of 200 · min^−1^, and mechanical ventilation was restarted with a FiO_2_ of 1.0 at a respiratory rate of 50 · min^−1^. Thirty seconds after starting chest compressions, an intravenous bolus of 0.02 mg · kg^−1^ adrenaline (epinephrine) was administered to all animals via the femoral line. External defibrillation with 5 J (Zoll MSeries, Zoll Medical Corporation, Chelmsford, MA, USA) was attempted after 3 minutes of CPR and repeated up to three times. If ROSC came to nothing, the procedure (chest compressions, administration of adrenaline) was repeated for 1 minute before defibrillation (again up to three times) was attempted. This cycle was repeated up to three times. Spontaneous cardiac rhythm in conjunction with a rise in MAP to greater than 50 mmHg affirmed ROSC. During the first hour after ROSC, all groups received 100% oxygen before FiO_2_ was reduced to 0.3. Animals of the MTH -group were cooled immediately after ROSC for six hours and reached the target temperature of 32–34 °C within 15 minutes. Gradual rewarming was achieved within one hour using a heating pad. Animals of the MTH + iNO group were treated identically and additionally received 20ppm iNO for five hours, starting one hour after ROSC. The control group did not receive any additional treatment and was kept normothermic (37.0–37.5 °C). Seven hours post ROSC animals were weaned from the ventilator and returned to their cages.

### Measurements

Ischemia time represents the sum of the duration of VF (10 minutes), CPR (three minutes) and the additional time needed to achieve ROSC (at most three additional minutes of CPR). A multichannel data acquisition system (Power Lab, AD Instruments, Spechbach, Germany) was used to record Heart rate, MAP, DAP and end-tidal CO_2_ continuously. At baseline (BL), 30 minutes (PR 30 min), one hour (PR 1 h), four hours (PR 4 h) and six hours (PR 6 h) after ROSC, arterial blood samples were taken. Arterial oxygen (p_a_O_2_) and carbon dioxide (p_a_CO_2_) partial pressures as well as haemoglobin, hydrogencarbonate, pH-values, and lactate levels were measured using a conventional blood gas analyzer (ABL700, Radiometer Copenhagen, Denmark).

Additional blood samples were taken at BL, PR 4 h, and PR 6 h, in standard serum gel collection tubes, containing coagulation activator gel (S-Monovette, Sarstedt inc., Nümbrecht; Germany), an allowed to clot for 30 minutes before centrifugation at 2500 × G at 4 °C for 10 minutes. The supernatant was immediately stored at −80 °C for subsequent analysis of tumor-necrosis-factor-alpha (TNF-α) using a rat ELISA (Rat TNF-alpha Quantikine ELISA Kit, RTA00, R&D Systems, Wiesbaden-Nordenstadt, Germany).

### Neurological deficit score

Performed by investigators blinded to the animals’ treatment, neurological performance was evaluated daily on the seven days following CPR. The apllied neurological deficit score (NDS) was previously established in an asphyxia CA model^22^, and validated by our group in this model^21^. Consciousness, respiration, cranial nerves, motor and sensory function and coordination were graded depending on the severity of the impairment and given a score, which ranges from 0 (worst neurological impairment) to 500 (no neurological impairment).

### Histopathology

After completion of the seven days NDS testing phase, rats were re-anaesthetized as described above. A midline thoracotomy was performed and the animals were transcardially perfused with 100 ml of NaCl 0.9%.

#### Neurohistopathology

Afterwards, the brains were carefully explanted and transsagitally cut in half. 4% buffered paraformaldehyde was used to postfix the right hemispheres. After fixation, a custom made brain matrix was used to cut the hemisphere in eight standardized coronal slices, each at a thickness of 2 mm. As regions of interest (ROI), the neocortex, hippocampal CA 1 and anterior and posterior CA 3/4 sectors, as well as basal ganglia were analysed by an experienced neuropathologist blinded to the animals’ treatment. Hematoxylin and eosin (H&E) staining was performed to assess neuronal damage in terms of hypereosinophilic and/or shrunken cytoplasm, pyknotic nuclei or complete cell loss. Immunohistochemical staining of adjacent sections with anti-Neuronal nuclei (mouse monoclonal Ab, Millipore/Chemicon International, Cat No MAB377) was additionally carried out to corroborate H&E findings since neuron-specific nuclear protein (NeuN) immunoreactivity is widely being used as a biomarker for assessment of the functional state and viability of many postmitotic neuronal cell types including rodent hippocampus^23,24^. Although reduced anti-NeuN labeling can be observed in different pathological conditions^25,26^ and should not be taken as an unequivocal evidence of neuronal death^27^ decrease or loss of NeuN immunoreactivity is still a helpful parameter to indicate neuronal metabolic perturbations and predict cell degeneration^26^ especially when being correlated with classical signs of neuronal desintegration according to H&E stain. As previously established in a modified model of CA and CPR^21^, a neuronal damage index (NDI) was semiquantitatively assessed. The number of damaged neurons was assessed in multiple ROI windows (neocortex, basal ganglia) and the whole CA1/ CA3/4 band^28^, to avoid unrepresentative results due to inhomogeneous distribution of viable or injured neurons in the ROIs. Finally, the proportion of neuronal cells showing shrunken and/or hypereosinophilic cytoplasm (H&E staining) in combination with a significant decrease or loss of NeuN-immunoreactivity was determined and summarized as follows: 0–5% = 1, 5–10% = 2, 10–20% = 3, 20–30% = 4, 30–40% = 5, 40–50% = 6, 50–60% = 7, 60–70% = 8, 70–80% = 9, 80–90% = 10, 90–100% = 11.

#### Myocardial Histopathology

Hearts were removed after transcardial perfusion and bisected in transversal direction. Remnants of atria and the aortic arch were removed, and the sections were fixed in 4% buffered formalin for at least 24 hours prior to embedding in paraffin. In total, 10 standardized cross sections of the ventricular parts at a thickness of 2 µm each were taken per heart. A previously described standard protocol for Luxol fast blue (LFB) staining, and counterstaining with nuclear fast red was apllied^29^. Using ImageJ software (version 1.46r, National Institutes of Health, Bethesda, MD, USA), planimetric analysis was performed by two blinded investigators, and validated by an experienced pathologist. LFB positive controls (infarcted rat hearts) were used to calibrate the software to a threshold value for the detection of blue color (blue transverse banding and diffuse blue myocytes) to distinguish myofibrillar degeneration (LFB, blue), and normal tissue (nuclear fast red, purple). The degree of tissue damage was expressed as percentage of blue-staining from the total area, by averaging the values of the planimetric analysis of the two experimenters.

### Statistical analysis

Normal distribution of the results was confirmed using the D’Agostino-Pearson omnibus and the Shapiro Wilk-Test. The results are expressed as mean ± standard error of the mean (SEM) unless otherwise mentioned. One-way ANOVA with Tukey correction was applied to test parameters at distinct time points for significant differences between the groups. To test time-dependent variables for significant differences between the groups, a two-way ANOVA with Tukey correction for multiple comparisons and a confidence interval of 95% was used. In not normally distributed data, group comparisons were performed using a non-parametric Kruskal-Wallis test, followed by pairwise post-hoc testing with alpha correction. Statistical analyses were performed using IBM SPSS statistic 24 and GraphPad Prism 6.04 (GraphPad Software Inc.) with two-tailed hypothesis. A p-value ≤ 0.05 was regarded as indicator for statistical significance in all cases. A power and sample Size calculation was performed using PS Power and Sample Size Calculation version 2.1.31 software by Dupont and Plummer^30^, based on data derived from pilot experiments.

### Data availability

The authors comply with the publication’s requirements for sharing materials.

## Results

### Baseline

**Figure 1:**
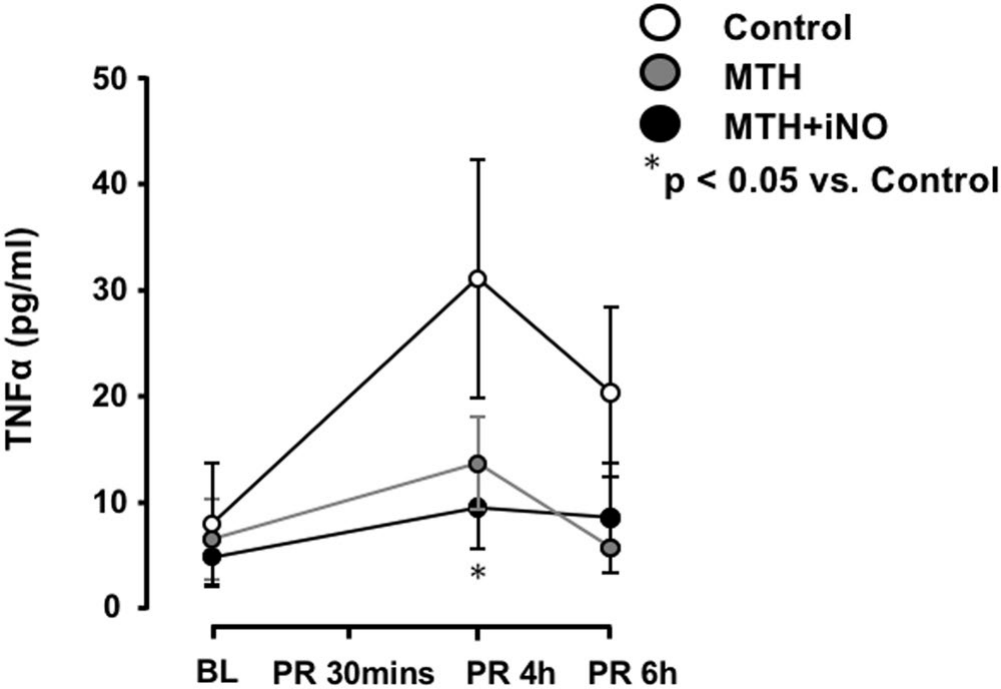
Alterations in TNF α levels before CA and after return of spontaneous circulation (ROSC). The significantly attenuated inflammatory response in the MTH + iNO group in comparison to Controls four hours post ROSC was reversed at hour six. *p < 0.05 vs control; mean ± SEM.

No significant differences were observed regarding haemodynamics, variables of gas exchange and lactate concentrations between MTH, MTH + iNO treated animals and Control at baseline (Table 1, Fig. 1).

**Table 1:** Physiologic data.

		BL	PR 30 mins	PR 1 h	PR 4h	PR 6h
HR (bpm)	Control	383 ± 8	340 ± 37	364 ± 18	364 ± 12	364 ± 7
	MTH	423 ± 5	299 ± 16	358 ± 17	334 ±	9341 ± 8
	MTH + iNO	405 ± 19	299 ± 16	315 ± 16	325 ± 14	346 ± 18
MAP (mmHg)	Control	140 ± 7	90 ± 3	91 ± 4	103 ± 5	101/23
	MTH	154 ± 4	93 ± 8	98 ± 10	120 ± 5	127*/17
	MTH + iNO	140 ± 8	88 ± 5	86 ± 5	107 ± 9	120/16
Hgb (g/dl)	Control	13.8 ± 0.3	15.6 ± 0.3	15.2 ± 0.2	13.8 ± 0.4	13.6 ± 0.5
	MTH	14.2 ± 0.2	15.9 ± 0.3	15.7 ± 0.4	14.6 ± 0.4	13.6 ± 0.4
	MTH + iNO	14.1 ± 0.3	14.5† ± 0.5	15.3 ± 0.3	14.2 ± 0.4	13.4 ± 0.4
HCO3^−^	Control	26.2 ± 0.8	18.3 ± 1.4	21.1 ± 2.0	23.4 ± 1.5	22.9 ± 1.8
	MTH	28.0 ± 1.0	15.3 ± 0.9	21.0 ± 0.9	23.6 ± 0.7	22.9 ± 0.5
	MTH + iNO	27.7 ± 0.5	14.8 ± 1.9	18.3 ± 1.6	22.4 ± 0.7	22.3 ± 0.8
pH	Control	7.4 ± 0.0	7.2 ± 0.0	7.3 ± 0.1	7.4 ± 0.0	7.4 ± 0.0
	MTH	7.4 ± 0.0	7.1 ± 0.0	7.3 ± 0.0	7.4 ± 0.0	7.4 ± 0.0
	MTH + iNO	7.4 ± 0.0	7.1 ± 0.1	7.2 ± 0.1	7.4 ± 0.0	7.4 ± 0.0
Lactate (mmol/L)	Control	0.9 ± 1.5	4.1 ± 0.5	2.6 ± 0.7	1.1/0.5	1.2/1.4
	MTH	0.8 ± 0.1	5.4 ± 0.8	1.9 ± 0.2	0.6*/0.3	0.7*/0.2
	MTH + iNO	1.0 ± 0.1	5.9 ± 1.6	3.1 ± 0.9	0.9/0.6	0.5*/0.3
PaO_2_ (mmHg)	Control	124 ± 14	237 ± 60	207 ± 69	148 ± 11	119 ± 11
	MTH	108 ± 11	302 ± 59.1	297 ± 58	146 ± 13	154 ± 10
	MTH + iNO	99 ± 8	280 ± 42	277 ± 43	126 ± 6	132 ± 9.1
PaCO_2_ (mmHg)	Control	48 ± 2	53 ± 3	52 ± 9	37 ± 3	38 ± 0.0
	MTH	48 ± 1.2	58 ± 8	46 ± 1.7	40 ± 3	38 ± 4
	MTH + iNO	47 ± 1.5	51 ± 3.7	47 ± 3	35 ± 2	39 ± 2.1
Temperature (°C)	Control	37.4 ± 0.1	37.2 ± 0.0	37.4 ± 0.1	37.3 ± 0.0	37.3 ± 0.0
	MTH	37.4 ± 0.1	32.8* ± 0.2	33.1* ± 0.2	33.0* ± 0.3	33.0* ± 0.3
	MTH + iNO	37.3 ± 0.1	32.7* ± 0.2	33.1* ± 0.2	33.1* ± 0.2	33.1* ± 0.2

Physiologic data for control, MTH and MTH + iNO treated animals at baseline and after cardiopulmonary resuscitation. Temperature indicates body temperature measured using an oesophageal probe. *p < 0.05 vs. control, ^†^p < 0.05 vs. MTH; mean ± SEM, except not normally distributed Lactate at PR 4h and PR 6h and MAP at PR 6h. HR heart rate, MAP mean arterial pressure, Hgb haemoglobin, HCO3^−^ hydrogencarbonate, PaO_2_ arterial oxygen tension, PaCO_2_ arterial carbon dioxide tension, PR time post-resuscitation.

### Measurements

Ischemia time was comparable in all groups: MTH + iNO: 867 ± 70 s vs. MTH: 782 ± 1 s vs. Control: 824 ± 28 s (MTH + iNO vs. MTH p = 0.432, MTH + iNO vs. Control p = 0.804, MTH + iNO vs. Control p = 0.816).

Post-resuscitation MAP was significantly lower than at baseline in all groups. While MTH treated groups recovered more quickly from haemodynamic depression, MAP remained decreased throughout the observation period in control animals. This reached statistical significance at PR 6 h, when animals of the MTH group presented with a significantly higher MAP than controls. Tissue ischaemia, as indicated by a marked increase in lactate levels 30 minutes post-resuscitation, was present in all groups. However, only MTH treated groups presented with a marked decrease in lactate levels four and six hours post resuscitation, being significant for MTH treated rats at both time points and when iNO was added at hour six in comparison to control animals (Table 1).

**Figure 2:**
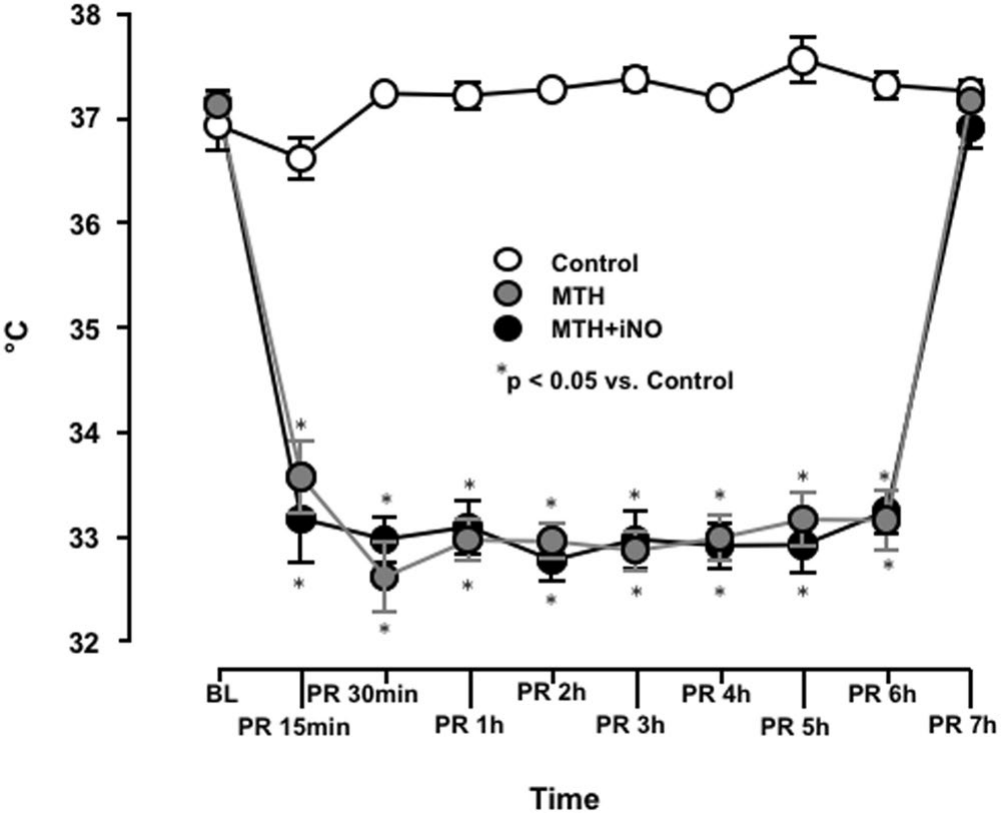
Experimental temperature control. Oesophageal temperature of 20 rats, either treated with normothermia (control, n = 7), mild therapeutic hypothermia within 15 minutes post ROSC for six hours (MTH, n = 6) or 20ppm iNO (MTH + iNO, n = 7) for five hours, starting 1 hour post ROSC in combination with MTH to baseline (BL) and post resuscitation (PR). *p < 0.05 vs control; mean ± SEM.

In the normothermic control group, oesophageal temperature was tightly around 37.3 °C during the whole observation period (Fig. 2, Table 1). All animals of the hypothermic groups reached MTH within 15 minutes. Oesophageal temperature was tightly around 33.0 °C during MTH (Fig. 2, Table 1). All animals reached normothermia 7 hours post ROSC (Fig. 2).

Control animals exhibited an increased release of TNF-α post resuscitation, which was significantly less pronounced in the MTH + iNO group only at four hours post ROSC (Fig. 1).

### Neurological deficit score

**Figure 3:**
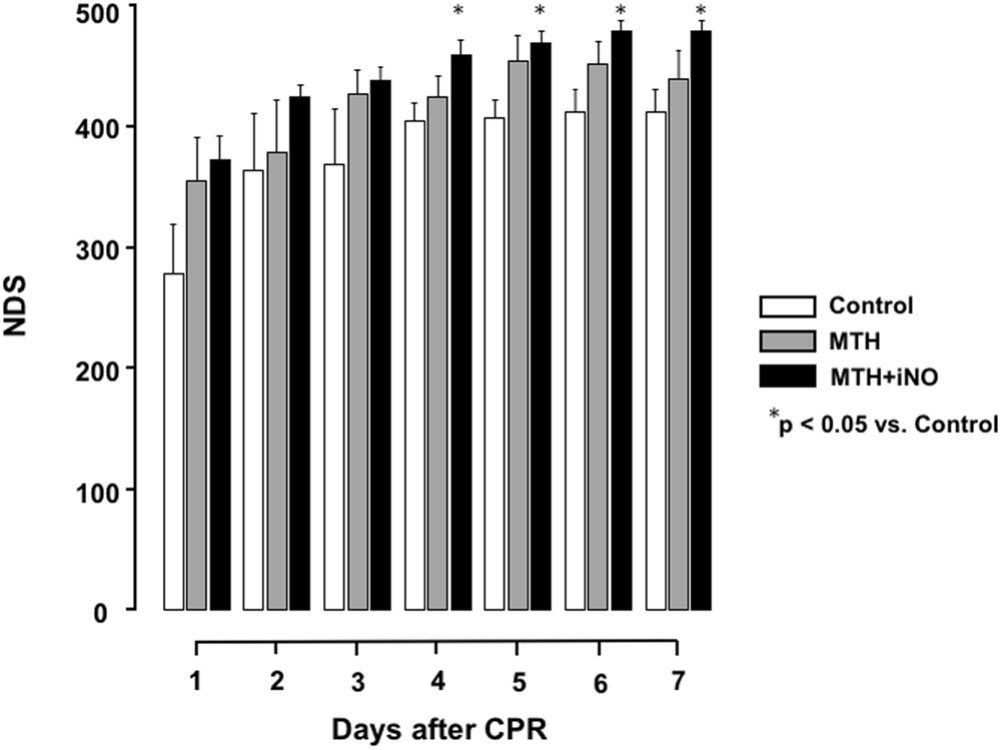
Neurological Deficit Score. Though animals of the MTH and iNO group presented with significantly improved neurological function on day four to seven post-arrest in comparison to Controls, no significant differences were observed in comparison to animals treated with MTH alone. *p < 0.05 vs. control; mean ± SEM.

We observed severe neurological dysfunction as measured with the NDS in all control animals during the seven days after CA and CPR. MTH markedly improved neurological function, however only to a statistical significant extent on days 4–7 after CA when MTH was augmented with iNO in comparison to the control group (Fig. 3).

### Histopathology

Neurohistopathological evaluation did not reveal any statistically significant differences between groups (Table 2).

**Table 2:** Neuronal damage index.

NDI	Neocortex	CA 1	CA 3/4	Basal ganglia
Control	1.00 ± 0.00	4.60 ± 1.86	1.00 ± 0.00	1.00 ± 0.00
MTH	1.00 ± 0.00	6.00 ± 1.46	1.00 ± 0.00	1.00 ± 0.00
MTH + iNO	1.17 ± 0.17	3.38 ± 1.54	1.60 ± 0.40	2.00 ± 0.00

Conventional hematoxylin/eosin (H&E) and Mouse anti-Neuronal Nuclei (NeuN) staining was performed. A neuronal damage index (NDI) was semiquantitatively assessed for all regions of interest. The proportion of neuronal cells showing shrunken and/or hypereosinophilic cytoplasm (H&E staining) in combination with a significant decrease or loss of NeuN-immunoreactivity was determined and summarized in a score from 1(no/minimum damage) − 11 (maximum damage). Histopathological evaluation did not reveal any statistically significant differences between groups; mean ± SEM.

**Figure 4:**
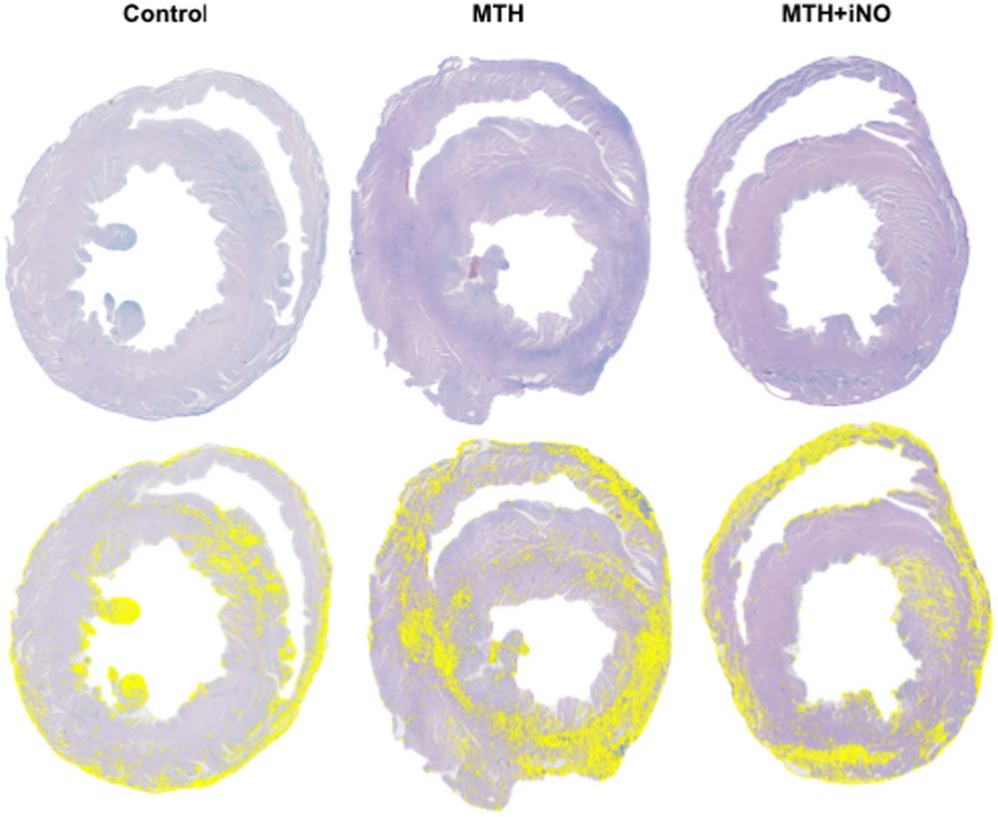
Myocardial Histopathology. Successful CPR resulted in cardiac myofibrillar degeneration, detected by luxol fast blue staining (LFB). Representative photomicrographs of heart cross sections with LFB and nuclear fast red counterstaining on day 7 post ROSC of a control, an iNO and MTH + iNO treated animal. The planimetry analysis with imageJ software discriminates between myofibrillar degeneration (LFB blue staining, yellow markers) and undamaged tissue (nuclear fast red counterstaining, purple). No significant differences were observed between groups (MTH 64 ± 5, MTH + iNO 54 ± 3, Control 57 ± 6, MTH vs Control p = 0.96, MTH + iNO vs Control p = 0.91, MTH vs MTH + iNO p = 0.32).

We observed that CA and CPR caused myocardial damage in all rats without significant differences between groups as demonstrated by LFB staining (Fig. 4).

## Discussion

Our results demonstrate that only the combination of MTH and inhalation of 20ppm iNO, but not MTH alone, improves functional outcome in rats on days four to seven, when administered for five hours with a delay of one hour following CA. Though animals of the MTH and iNO group presented with significantly improved neurological function on days four to seven post-arrest in comparison to controls, no significant differences were observed in comparison to animals treated with MTH alone (Fig. 3). While we observed a faster haemodynamic recovery of MTH treated groups after ROSC resulting into significantly reduced lactate levels six hours post ROSC in both MTH groups in comparison to controls, we did not observe statistically significant differences between MTH treated groups (Table 1). The significantly attenuated TNFα response in the MTH + iNO group in comparison to controls four hours post ROSC was reversed at hour six (Fig. 1). These findings clearly extend findings of our recent study^17^: While our previous findings show, that short term iNO administration **during** CPR increases resuscitation success and improves neurological outcome, the current study demonstrates, in a clinically more relevant scenario of **post** CA iNO administration, that iNO combined with the gold standard MTH does not improve cerebral and myocardial outcomes in a manner exceeding the effect of MTH alone.

To date, post CA TTM with targeted body temperatures between 32–36 °C is recommended by the 2015 American Heart Association Guidelines for Cardiopulmonary Resuscitation and Emergency Cardiovascular Care, and remains the only neuroprotective treatment after CA that is endorsed by international guidelines^31^. However, a large population of CA victims resuscitated from a nonshockable rhythm does not seem to profit from MTH. Treatment strategies to further improve outcomes after CA beyond targeted temperature management are highly desirable.

Interestingly, Kida and colleagues demonstrated that inhalation of nitric oxide at 40 or 60ppm NO starting up to two hours after ROSC and continued for 24 hours improves the survival rate after CA/CPR in mice above and beyond MTH alone. Additionally, they could show in their study, that inhaled nitric oxide augmented MTH, exerts brain protection exceeding the effect of MTH alone^19^. This is in contrast to our study. The lack of a beneficial effect of inhaled nitric oxide above the effect of MTH alone, might be due to several aspects: Initiation and duration of MTH differed between studies fundamentally. Kida used delayed MTH for 24 hours in contrast to immediate MTH for only 6 hours in our study. However, Jia and colleagues could show in an asphyxial cardiac arrest model that immediately induced MTH maintained for six hours, resulted in better neurological outcome when compared to delayed induction and 12-hour maintenance of MTH^32^. None the less, in this regard, Kida’s model seems to be clinically more relevant. While immediate MTH is easily instituted in the lab (Fig. 2), it may substantially differ from clinical practice, which is at best goal temperature within one hour. Furthermore, Kida and colleagues observed outstanding effects of iNO at a concentration of 40 and 60ppm, but not 10, 20 or 80ppm, starting 30 minutes up to two hours (40ppm) after ROSC and continued for 24 hours. In the present study, we used shorter periods of lower concentrated iNO, which might be an additional reason for the lack of an additive beneficial effect of NO inhalation in MTH treated rats. However, in our latter study the effect of 20ppm iNO was superior to 40ppm short term iNO administration during CPR until 30 minutes thereafter^17^. While Kida and colleagues used mice, we examined rats. While species-based difference might be significant, preceding studies demonstrated beneficial effects of iNO in the setting of CA/CPR in mice^16^, rats^17^ and swine^18^. Additionally, Kida and colleagues employed a model of potassium induced CA, while we examined a VF model, which mimics a clinically more relevant scenario. In our study, the marginal beneficial effects of iNO and MTH on the neurological outcome were not reflected in histopathological results (Table 2). Similar findings were observed in previous studies^16^.

I/R injury is related to activation of neutrophils with release of pro-inflammatory cytokines^33^. The activated cascades of immunological and coagulation pathways after ROSC, as part of the post-cardiac arrest syndrome, resemble severe sepsis with elevated serologic markers^8,34^. Inflammatory cytokines have been implicated in myocardial and brain dysfunction in the early post CA period. In particular, TNF-α levels increase shortly after CA, are inversely correlated with myocardial function^35,36^ after CA and are predictive of early death. TNF-α also seems to play a crucial role in CA induced neuroinflammation^37^ and is shown to be temporo-regional specific^38^. While TTM does not appear to modify levels of the inflammatory markers interleukin (IL)-1β, IL-6, TNF-α, IL-4, IL-10, C-reactive Protein (CRP) and Procalcitonin (PCT)^39^, recent studies suggested salutary effects of iNO on markers of inflammation and oxidative stress at least in patients undergoing cardiac surgery^40^. Data from Minamishima and colleagues suggest that inflammation attenuated by iNO might play a crucial role in brain protection by preventing the induction of TNF-α and IL-6 expression in the brain 24 hours after CA/CPR in mice via soluble guanylate cyclase -dependent mechanisms^16^. In addition, a recently published study from our group revealed a significantly attenuated release of TNF-α in iNO treated animals following CA and CPR^17^. In the present study, a significantly attenuated TNF- α release in the MTH + iNO group could only be observed in comparison to controls at four hours post ROSC, which was reversed at hour six (Fig. 1). Differences in TNF-α levels earlier than 1 hour post CA might have stayed undetected. However, in our previous study we did not find significant differences between groups 30 minutes post ROSC^17^.

Although iNO is believed to exert beneficial effects in remote organs, we did not observe significant differences between groups in myocardial damage assessed on day seven in the present study (Fig. 4). This is in line with other milder animal models of CA/CPR, where histological cell death is unlikely be found^41^. However, in terms of better functional outcome, myocardial protection was found by Dezfulian and co-workers when an injectable NO donor was given at the initiation of CPR^42^ and by Minamishima and colleagues when iNO was administered after CA/CPR in mice^16^. In the present study, early functional cardiac outcome has not been evaluated, e.g. troponin release or cardiac function assessed by echocardiography. Additionally, regional TNF-α expression of the heart or brain has not been assessed.

We recognize several further limitations in the interpretation of our findings. Data were obtained from healthy animals, and translation of results to humans, with pre-existing diseases, has always to be made with caution. The effect of iNO in this model has not been assessed independently, but only as an adjunct to the existing standard of care- MTH. Additionally, histopathological evaluation of the brains did not reveal any significant differences between groups on day seven. However, as neuronal death evolves at least between days three and day fourteen^43^, it might well have been that seven day outcome may not represent the final stage of histological damage. Furthermore, the design of the study does not allow for any conclusions to be drawn as to the most appropriate concentrations of iNO or whether increased duration of ventilation with iNO or TTM would provide a greater degree of protection. However, in a previous study from our group the effect of 20ppm iNO was superior to 40ppm iNO during CPR until 30 minutes thereafter^17^. Additionally, we cannot exclude differences in body temperatures beyond weaning from the respirator. At last, functional neurological outcome was only monitored for up to seven days after CA, and control animals may have recovered when allowed for a longer period of time.

## Conclusion

While only the combination of MTH and iNO improved neurological function in a statistically significant manner in comparison to controls on days 4–7 after CA, there was no significant difference between groups treated with MTH and MTH + iNO.
